# Existential dimensions of the lived experiences of transgender youths 13-16 years in a cisnormative Swedish context

**DOI:** 10.1080/26895269.2024.2440864

**Published:** 2025-01-06

**Authors:** Kristiina Tyni, Matilda Wurm, Anna Sofia Bratt

**Affiliations:** a Department of Psychology, Linnaeus University, Växjö, Sweden; b School of Behavioral, Social and Legal Sciences, Örebro University, Örebro, Sweden

**Keywords:** Existential, gender identity, LGBTQ, qualitative research, teen

## Abstract

**Background:**

Transgender and gender diverse (TGD) youth face existential challenges in navigating their genders in a cisnormative world that often misrecognizes or denies them.

**Aim:**

This qualitative study aimed to deepen the understanding of existential dimensions of the participants’ lived experiences related to gender, within a cisnormative context.

**Methods:**

A reflexive thematic analysis was conducted on the lived experiences of nine Swedish binary transgender youths, aged 13-16.

**Results:**

Two main themes were developed: 1) “Disorientation and existential challenges: navigating self-recognition in a non-inclusive world,” with subthemes *Can I exist in the world*’ and *Can I exist in my body?;* and 2) “Relational responsibility: emotional work to be recognized,” with subthemes *Taking responsibility for my safety; Responsibility for others*—*facilitating recognition*; and *Reorientation and the power of recognition.*

**Discussion:**

Drawing on Ahmed’s Queer Phenomenology and Honneth’s Theory of Recognition, the study highlighted how limited awareness or denial of gender diversity shaped participants’ struggles to orient themselves in a cisnormative society. Living authentically required significant emotional labor in navigating relationships, reflecting an ongoing process of coping and a deep need for emotional, communal, and legal recognition.

**Conclusion:**

By exploring the existential dimensions of participants’ lived experiences within cisnormativity, this study underscored the power of (mis-)recognition and the emotional labor TGD youth undertake to create livable spaces for authentic lives. A holistic understanding of their experiences can guide professionals, parents, and others in providing better support.

## Introduction

There is a growing body of literature on the lived experiences of transgender and gender diverse (TGD) youth (Jessen et al., 2021; Saltis et al., 2022; Tyni et al., 2024a, 2024b). Research has shown the crucial role of recognition and belonging for the mental health and well-being of TGD youth (Austin et al., 2022; Kelley et al., 2022; McGowan et al., 2022; Russell et al., 2018). However, there remains a lack of explicit focus on the existential dimensions of TGD youths’ lived experiences. This study addresses this gap by exploring the existential dimensions of nine Swedish transgender youths’ lived experiences within a cisnormative context.

Existential phenomena in lived experiences are often explored within the caring sciences from a phenomenological lifeworld perspective, rooted in Husserl’s philosophy and further developed by thinkers such as Merleau-Ponty (Lundvall et al., 2019). Similarly, Ahmed’s Queer Phenomenology (2006), used in this study, builds on classical phenomenology but incorporates queer and intersectional perspectives, focusing on the experiences of individuals who deviate from societal norms.

In research with adult TGD individuals there are a few studies more explicitly underlining existential dimensions. For example, a quantitative study found that transgender adults reported higher scores on measures of managing existential concerns, such as suicidal thoughts, lower meaning of life, and existential isolation compared to cisgender participants (Horner et al., 2023). Furthermore, lacking support for their gender expressions predicted greater post-traumatic stress, whereas support for their gender expressions predicted greater meaning of life and lower existential isolation (i.e. feeling lonely in one’s subjective experience, Pinel et al., 2022) in transgender participants (Horner et al., 2023). A qualitative study revealed that TGD adults engaged extensively in existential, relational, and reflective work to create a livable life where they could find moments of relief from the pressures of minority stress (Lundberg et al., 2024).

Meyer’s minority stress model (Meyer, 2003) and its further developments, such as the addition of microaggressions (Frost & Meyer, 2023; Nadal et al., 2016) have been used extensively to understand health disparities in sexual- and gender minority youth (Bränström, 2019). External and internal minority stressors such as experiences of gender-based bullying and internalized transphobia, as well as microaggressions—the small, everyday actions or comments that invalidate or make transgender persons feel invisible—have been shown to have a negative impact on mental health and well-being of TGD youth (Austin et al., 2022; Chodzen et al., 2019; Conn et al., 2023; Eisenberg et al., 2019; Kelley et al., 2022; Russell et al., 2018; Veale et al., 2017). This stress, stemming from their stigmatized minority status, adds to the general stress all youth can face in finding their identity and managing increasing demands at school and in social arenas (Wurm et al., 2024). To manage these minority stressors, TGD youth employ diverse coping strategies, such as seeking social support or hiding their gender, depending on context, timing, experience and maturation (Budge et al., 2018; Kelley et al., 2022; Saltis et al., 2023). However, the continuous work TGD individuals do in their daily lives, is still underexplored in research with TGD youth. In our qualitative systematic review on the experiences of TGD youth 18 years or younger (Tyni et al., 2024a) we were inspired by the concept of *emotional labor* (Hochschild, 1979) in line with recent Swedish research (Linander et al., 2019; Lundberg et al., 2024). Emotional labor for TGD individuals involves managing their emotions and gender presentation to navigate societal expectations, avoid discrimination, and maintain social (and professional for adults) harmony. This can include suppressing frustration or discomfort in response to misgendering, educating others about their identity, or emotionally preparing for rejection. The emotional toll of this labor, often invisible and unacknowledged, can lead to exhaustion and stress, as TGD people balance authenticity with the demands of a cisnormative world (Lundberg et al., 2024). Where coping (that is, behaviors we do to manage stressful events) mostly refers to managing separate incidents (Folkman & Lazarus, 1980), emotional labor includes the ongoing, daily work TGD individuals do to secure recognition and acceptance. Even prepubertal TGD children from 4 years old have been shown to display this kind of ongoing work related to their gender identities, emphasizing the challenges that even TGD youth with parental support must cope with when growing up in cisnormative contexts (Tyni et al., 2024b).

To get a deeper understanding of the existential dimensions of the studýs transgender participants’ lived experiences within cisnormativity, we draw on Sara Ahmed’s (2006) Queer phenomenology and Axel Honneth’s (1995) Theory of recognition. While rooted in different philosophical traditions, both theories intersect in addressing existential concerns within broader societal structures.

Ahmed’s (2006) Queer phenomenology explores how minority people orient themselves in a world directed by social and cultural norms of whiteness and heteronormativity. Feelings of comfort versus discomfort, happiness versus anxiety can indicate how well one fits within a given context, for example, being black in a predominantly white context and/or queer in a heterosexual one. In a context where one does not fit in, queer orientations often involve navigating discomfort to find or create spaces where one feels more at home, grounded in a terrain where one can orient oneself (Ahmed, 2006). We believe that Ahmed’s queer phenomenological reasoning could illuminate the limitations and exclusions of cisnormative contexts and add a (queer) existential dimension to how the alignment or deviation from societal gender norms affect TGD youths’ lived experiences.

We also found Honneth’s intersubjective Theory of recognition—summarized as “love, rights, and solidarity” (Honneth, 1995)—relevant as he explores how social norms shape individual identities and how we can create conditions where people feel valued as human beings. Honneth’s three modes of recognition in the emotional, communal and legal spheres collectively form a theory of both individual development in a social context and social change in a historical context. We believe that these three modes of recognition collectively could illuminate TGD youths’ challenges on an existential level. A few studies involving adult transgender people have utilized Honneth’s theory, for example, to understand the importance of community connectedness and well-being (Ceatha et al., 2019) and to identify different legal models of identity recognition in comparing transgender rights in different countries (Aboim, 2022) but, as far as we know, it has not been used in studies with TGD youth. Honneth (1995) does not explicitly talk about children other than as receivers of familial love and care. Our standpoint, however, is that children have rights in accordance with the Convention on the rights of the child (1989)—which is also part of the law in Sweden. In addition, we recognize the vital importance of recognition for the mental health and well-being of TGD youth. Given this, we believe that Honneth’s thinking could be useful for discussing how TGD youth strive for and receive (or don’t receive) recognition. This is not just about viewing TGD youth as receivers of care, but also acknowledging them as active subjects and community members, whose rights and contributions should be valued and respected.

TGD youth often face significant challenges in just being themselves and expressing their gender identities. This qualitative study sheds light on the existential dimensions of nine Swedish transgender youths’ lived experiences within a cisnormative world, discussed through the lens of Ahmed’s Queer phenomenology (2006) and Honneth’s Theory of recognition (1995). In this study, “existential” refers to core aspects of the participants’ lives, including their gender identities, their sense of purpose and meaning, and their place in a cisnormative society. Examples include the significance of living authentically (Crosse, 2024) by expressing their true selves, or experiencing existential isolation (Pinel et al., 2022) in their subjective realities.

## Methods

### Epistemological stance and positionality

Our previous understanding of TGD youths’ experiences is based on the Minority stress model (Meyer, 2003), microaggressions (Nadal et al., 2012), and coping (Folkman & Lazarus, 1980). In this study however, we have aimed to expand our understanding by discussing our results through the theories of Ahmed’s (2006) Queer phenomenology and Honneth’s (1995) Theory of recognition. While the empirically grounded Minority stress model describes the effects of exposure on individuals, the (queer/feminist and social) critical theory frameworks of Queer phenomenology (Ahmed, 2006) and the Theory of recognition (Honneth, 1995) give additional tools to understand how the structural conditions of cisnormativity can shape TGD youths’ experiences.

Our research group consists of licensed psychologists with clinical experience of working with TGD people and/or doing research on TGD issues. We are all assigned females. Two of us have lived experiences of not being heterosexual and cisgender.

### Ethics

This study was conducted within a larger project focusing the lived experiences of a total of 19 transgender children and youths aged 4-16 years and their parents with an ethical approval granted by the Swedish Ethical Review Authority (Dnr 2020-01854). In this study only the data from the teens were included.

### Procedure

Families with minor children and youths identifying as some other gender (including non-binary identities) than the one assigned to them at birth were recruited to the larger study that the teens in this study were part of. An ad about the larger study was published through various online media channels, including Facebook groups for parents of trans children, Swedish LGBTQ organizations, a municipal site for LGBTQ youth activities, and the authors’ social media networks. To be eligible for the study, both the child/youth and at least one parent had to participate. Interested families contacted the first author (KT), and upon expressing interest, they received age-appropriate information by email and had the possibility to ask questions. The children/youths and their parent(-s) confirmed their participation by returning a signed informed consent form for each person. For all participants younger than 15 years, both legal guardians had to sign their child’s informed consent in line with Swedish research ethics. During the scheduled meeting, held at a date and location chosen by the youths, they received a verbal recap of the information before proceeding with the individual interviews. The parents were interviewed separately and the results from their data will be published elsewhere. In this study, only data from the teenagers were included.

### Participants and context

Nine transgender youths, aged 13-16 years at the time of interviews, participated in this study. The youths first expressed their trangender identities verbally to someone at varying ages - four participants did so at the age of three while five participants began from the age of 12 onward. However, this study primarily focuses on the participants’ experiences as teenagers. All participants had a binary identification of girl/trans girl (*n* = 2), or boy/trans boy (*n* = 7). Geographically diverse, the participants lived in various settings, including large cities, smaller towns, and rural areas across Sweden. Despite an ambition for intersectional recruitment, the youths were predominantly from white, middle-class families. Since all participants identified as binary genders, we use the term transgender when specifically referring to them, but use the term transgender and gender diverse (TGD) when referring to other studies to include a variety of concepts and data samples.

All participants had changed their names and pronouns, also in contexts outside their families, such as at school. A majority of the youths had also legally changed their names, but none were allowed to legally change their gender according to the Swedish jurisdiction at the time.

One participant had not been referred to gender-affirming care; one was in the process of getting referred; four had been referred and waited for their first appointment; and three had gone through the assessment procedure and had been diagnosed with gender dysphoria. At the time of interviews for this study, there were long waiting lists for gender-affirming care and uneven access across different regions, but a process of re-organizing the gender-affirming care to shorten the queues had begun.

None of the youths had yet been prescribed hormones due to a change of guidelines in Sweden (Socialstyrelsen, 2022) restricting hormonal treatment for people under 18 years. One participant had puberty blockers before new medical restrictions were implemented at the Karolinska hospital (Linander & Repka, 2021). When the family realized that their child would now have to wait many years for hormonal replacement therapy they decided to attempt to get prescriptions for hormones from gender-affirming care outside of Sweden. They had had a first online appointment.

### Data collection

The interviews were conducted by the first author (KT) between Oct 2020 and March 2023. Due to the Covid pandemic, interviews were postponed for two periods. Two families with teenagers who had previously sent informed consent did not answer new invitations for interviews sent out in May 2021 after the first break, so their reasons for not participating are unknown. However, at approximately the same time, in May 2021, the new restrictions of hormonal treatment in gender-affirming care were announced by the Karolinska hospital (Linander & Repka, 2021). One other family declined their participation due to negative consequences of the announcement for their youth’s mental health condition.

According to the wish of the participants in this study, the interviews took place in the families’ homes or digitally (Zoom). All meetings began with a verbal recap of the informed consent and a possibility to ask questions before conducting individual interviews. Interviews were tape-recorded, transcribed verbatim, and anonymized to protect youths’ confidentiality. The interviews lasted between 20 and 79 min (*M:* 47). The semi-structured interview questions were related to three areas: 1) name and pronoun, 2) gender identity, and 3) social areas, with flexible follow-up questions asking for specificity, examples, and/or clarification (see interview guide in Appendix 1).

### Data analysis

The interview data was analyzed through a reflexive inductive Thematic Analysis (Braun & Clark, 2021). KT first familiarized herself closely with the data by reading and re-reading transcripts and then, in a second step, coding the text sections relevant to the youths’ lived experience related to gender identity. In the third step potential themes and sub-themes were gathered and re-arranged. At this step of the analysis, we had discussions in the whole research group about the existential dimensions apparent in youths’ narratives. Therefore, during the final steps of the analysis, the theories of Ahmed’s Queer phenomenology and Honneth’s Theory of recognition, both of which appeared applicable and relevant to the participants’ experiences, informed our pre-understanding. However, these theories were not explicitly applied until the discussion of the results.

In a forth step the themes were refined using the initial codes and the entire dataset. Next, themes and sub-themes were labeled to convey their essence and citations were chosen from the data to give examples of how the themes are grounded empirically. In the sixth and final step the analysis was written into a comprehensive narrative. The analysis was conducted independently by KT, with regular discussions with ASB to ensure the trustworthiness and credibility of the data analysis.

## Results

The analysis resulted in two main themes where one focused on the felt disorientation and existential challenges, while the other focused on the relational work necessary for authentic living. The main themes have two and three sub-themes respectively. For an overview of main themes and subthemes—see Table 1.

**Table 1.** Main themes and subthemes from the analysis of TGD youth’s lived experience.

Main themes	Subthemes
Disorientation and existential challenges: navigating self-recognition in a non-inclusive world	Can I exist in the world?
	Can I exist in my body?
Relational responsibility: emotional work to be recognized	Taking responsibility for my safety
	Responsibility for others – facilitating recognition
	Reorientation and the power of recognition

### Disorientation and existential challenges: navigating self-recognition in a non-inclusive world

The participants faced existential questions about their place in the world and the value of life throughout their gender identity journeys. These challenges were heightened by the changes brought on by puberty, as well as by societal ignorance and their own and others’ limited understanding of gender diversity.

#### Can I exist in the world?

Many participants described how limited personal and societal knowledge about gender diversity made self-discovery difficult. Their existential experiences of who they were in regard to gender identity often clashed with their cisnormative expectations, leading to confusion or even shame, as this boy recounted:

All my life I’ve known I’m a boy, but I didn’t know the meaning of the word trans. I had no way to tell anyone because I didn’t even know that it existed, that it was something you could be, I just thought it was something I was thinking about and that it was impossible to be a boy, like: ‘why do I feel like a boy since I can’t be one’ […] So I was ashamed because it was so weird. (15-year-old boy)

Online resources served as a vital source of information and role models, helping participants reduce shame and feelings of existential isolation by showing them they were not alone in their experiences. Some participants noted that they first explored their sexual orientation before questioning their gender identity, which might have been influenced by the broader societal acceptance and understanding of sexual diversity compared to gender diversity. This greater familiarity with sexuality might have made it easier to process thoughts and feelings about sexual orientation than those related to gender identity.

Although experiencing their parents as generally supportive of their gender identities a few participants did encounter active denial within their families, such as a 16-year old boy who faced his father’s rejection: *“He told me several times that: ‘No, but you are a girl!’ and then he told me that I’m not normal because I don’t identify as my gender assigned at birth.[…] He didn’t accept me at all.”* What this youth described was not his father’s mere lack of knowledge or uncertainty about whether to be affirming or not, but rather a rejection of the existence of his child as a boy. Instances of active questioning or denial were however mostly described outside families.

The participants described instances of being harshly questioned or feeling invisible regarding their gender identities foremost at their schools, and many highlighted inadequate education about gender diversity. Some experienced their school environments as really lonely and alienating places until they were better received when changing schools. The youths underlined the important role of teachers for “setting the tone” regarding the school climate, but also of other grown-ups for conveying values to children generally. One participant, for example, described the parent of a classmate expressing that he confused the other children through his transgender identity. Another participant was questioned in his gender identity when a teacher at school reported the family to the social services for *“destructive gender experimentation.”* When the youths frequently had to manage invisibility and/or denial of who they felt they were, some even started to question the meaning of their existence. A 15-year-old boy articulated this existential struggle: *“I feel very sad and almost like there is no point in living if… if there isn’t anyone in the whole universe who sees me as myself.”*

The participants also expressed concern about societal resistance and hatred toward transgender individuals, impacting their everyday life and feeling of safety. They described the emotional toll of navigating these societal attitudes, sometimes using irony to distance themselves emotionally: *“Knowing that your existence is illegal in many countries and to be on the internet and see how many think you are-, well, ‘a creature against God’s design’ is super fun”,* said a 14-year-old boy. The youths were affected by their knowledge of negative attitudes toward transgender people to different degrees, from distinct situations, like having to plan which countries were safe to travel to for family vacations, to a more continuous every day cautiousness limiting their movements in society, for example being out on the town. Emotionally, the participants described feelings of fear and anger from being aware of the societal hatred, but also resentment and hopelessness. This kind of hatred, not dealing with a specific person or a concrete situation but rather societal attitudes or an abstract global cultural climate, was difficult for the youths to cope with.

#### Can I exist in my body?

The participants experienced heightened discomfort during puberty as their bodies increasingly diverged from their inner experience of who they were. The new tone of their voice, their changing body shape, or certain body parts and functions that developed or did not develop, could trigger painful emotional reactions connected to not recognizing themselves, feeling alien to their bodies and not being recognized by others the way they felt. For some, these feelings got so overwhelming that it triggered suicidal thoughts and led to self-injurous behavior when trying to cope with the pain on their own. As emotions intensified, the participants described that it got harder to bear their suffering silently, forcing them to reach out for help from family, councilors, and/or LGBTQ-organizations. Most youths also reached out to the gender-affirming care but were confronted with very long waiting times. Initially, they had to wait for a first appointment, sometimes for more than a year, and then there was further waiting time to undergo a diagnostic assessment process. If the assessment confirmed a youth’s gender dysphoria diagnosis, they had to wait again until they got at least 18 years old before a hormonal treatment could be considered. These waiting periods were emotionally challenging for most participants and felt *“like an eternity”,* as a 14-year old boy put it, highlighting the societal barriers they faced. While they longed to feel physically more congruent with, and to be able to present more like their gender identity socially, a consequence of the new medical age restrictions for hormonal treatment, was the risk of not being able to pass as their identified gender after going through puberty.

However, a few of the participants suffered less from gender dysphoria, maybe because they had come out quite recently and also described being well received. For them, the waiting felt emotionally easier and was even considered a possibility to really try out and be certain of their gender identities. On the other hand, they described not recognizing themselves in the dominant trans narratives, initially limiting their awareness of their gender identity, as shared by a 15-year-old girl:

I thought that I wasn’t ‘trans enough’, since all I read, at least in the beginning, sounded like you had to hate your body and not be able to get out of bed and have a lot, lot, lot of dysphoria and severe problems to be classified as trans. That was my first impression. (15-year-old girl)

Reflecting this girl’s experience, some participants doubted whether they were “*transgender enough*,” influenced by media narratives that often emphasized intense suffering. While it is important not to downplay the challenges faced, it is equally vital to represent the diverse experiences of transness shared by participants to create a more nuanced understanding. Still, despite varying degrees of suffering, all the youths were reminded of social exclusion by the need for official validation of their gender. As one 15-year-old boy noted: *“you have to get evidence of being trans”* to get recognition and basic rights that are automatically afforded to cisgender persons.

The necessity of obtaining a diagnosis for legal recognition further constrained the participants, creating dilemmas around identity disclosure in everyday situations. Not being able to change their legal identity without a diagnosis, the youths had to cope with a threat of having to disclose, defend, or explain their gender identity when showing their passport going abroad or just in daily life when showing their ID when fetching a parcel. This fear also impacted participation in enjoyable activities, such as swimming or going to the gym with no gender-neutral facilities. Two youths also described how their sports organizations required a diagnosis for team inclusion corresponding to their gender identities.

### Relational responsibility—emotional work to be recognized

The participants did a lot of emotional work by taking responsibility for themselves related to safety and to facilitate for others to recognize them. Being recognized in their gender identities on the other hand was associated with joyful feelings and a sense of belonging.

#### Taking responsibility for myself—can I be safe?

The participants described a heightened awareness of every day situations that could threaten their safety. Many had experienced gender-based harassment and bullying, particularly in school settings where areas with less adult presence felt the most unsafe:

Peers like to throw things at me and my friend in the corridors. We have got half full Coca-Cola bottles thrown at us when we are standing by our lockers, roasted corn, candy, erasers, several pens. You don’t feel very safe by the lockers- (15-year-old boy)

In line with this boy who felt worried about attending school, other participants described how the experiences of being bullied became a nagging worry over time about new incidents, affecting their mental and physical health in the form of depression, stomach aches, sleep disturbances, and difficulties attending classes. One participant refrained from expressing her gender identity, avoiding skirts or makeup because of her peers’ negative reactions toward others who deviated from norms. She blamed herself for “*lacking the courage*” to express herself, without recognizing the influence of societal norms shaping her peers’ attitudes. Similarly, many participants described suppressing their gender expressions or ignoring harassment to avoid drawing attention to themselves. These strategies often resulted in feelings of shame, guilt, and sadness, leading to social withdrawal and a lack of needed support.

The emotional toll of bullying also led the participants to conceal their gender identities in certain contexts. For example, one boy denied being transgender at a new school to avoid a repetition of the bullying he had faced previously. Another participant, working in a traditionally male-dominated job, hid her gender identity to avoid possible harassment and also feared losing privileges like technically challenging assignments and camaraderie from male colleagues. Her experience highlights the impact of cisnormative gender roles.

The participants described many ways to forsee situations and prepare themselves to minimize possible exposure, such as seeking information about gender-neutral facilities in advance or being cautious in public spaces, as described by a 14-year-old boy:*“The fear that someone can actually attack you for what you are, or just be very rude, is always there. I might not go around the streets telling everyone ‘I’m trans’. I might keep quiet about that.”*

Ultimately, these constant safety concerns affected participants emotionally and behaviorally, while also limiting their living space and their ability to move freely and authentically in the world.

#### Responsibility for others—facilitating recognition

The participants who came out as teenagers described mixed emotions: confidence in their parents’ support alongside worry about upsetting them. They often took responsibility for others’ reactions, striving not to trouble, burden, disappoint, or worry their families. Some felt the need to be absolutely certain about their gender identity before coming out, aiming to avoid causing distress by *"making a mistake,"* as one youth put it. Another participant initially told his mother he was nonbinary, believing it would be easier for her, despite identifying as binary transgender:

Then I actually told her the truth: ‘In fact I’m a trans boy and have always felt that way, but I thought it might be easier for you to-, if I was nonbinary’. That was the day after. She was still very shocked and, well, she had never experienced anything like that before, so I gave her a couple of weeks. I just told her, and then I left it for a couple of weeks. Then I came back to her and said: ‘Hi, can we talk some more about this. I have been thinking that I might want to be called [name] and I want to buy a binder so I look more flat’, and it took-, I gave her another couple of weeks and then she came-, she became my number one supporter when she got used to it. (15-year-old boy)

A 15-year-old girl whose father asked how she was doing after coming out, told him she was okay even though she was not: *"I don’t want to burden others with my problems."* Several participants reflected on their experiences, emphasizing the importance of talking to someone while questioning their gender identity. Many wished they had opened up sooner, noting how sharing their feelings had been positive. The participants’ efforts to manage their confusion and distress in silence deepened their vulnerability and created a sense of existential isolation.

While most participants had positive experiences with family, friends, and schools adapting to their name and pronoun changes, not everyone shared this experience. One boy, for example, was bullied at his previous school, where his pronouns were not respected. After changing schools, he found friends who knew him as socially transitioned from the start. Because he "passed" well, he decided not to disclose his transgender identity to peers, except to school staff for administrative reasons. He preferred to be known for who he was as a person, rather than being viewed through stereotypes about transgender people.

Many participants showed a strong sense of responsibility for others, displaying patience and understanding toward those who misgendered them. It was easier to cope when people demonstrated good intentions by correcting themselves or apologizing. However, being repeatedly misgendered by the same person—especially by familiar teachers or staff at leisure activities—was emotionally painful:

I mostly get irritated [when people say the wrong pronoun]. But when people who often say wrong, either on purpose or just because they are bad at learning, I get more-, well, then it gets harder. I have a teacher, my mentor, she is still really lousy at it, and I came out in the end of 8th grade, so she has had more than half a year to learn but she is still really bad at it. But when my parents-, they really try, so when they say it wrong it’s only by mistake. (15-year-old girl)

One participant struggled deeply with hurtful experiences in church, where personnel repeatedly failed to correct themselves despite many reminders. Leaving the church, however, was not a viable option for him, as it would mean losing his close-knit community of friends. This painful dilemma, of having to choose between being continuously misgendered or being lonely, was echoed also by other participants in relation to cisgender friends in their groups:

But I also have many friends who are not trans, and it feels like many of them don’t really see me as a boy, but rather as a girl who uses he-pronouns and that really hurts. At the same time, I have no choice but to hang out with them, because if I had shown them-, they are part of my friend-group and then I wouldn’t have any friends at all, so I just kind of have to get used to it, I have to take it. (15-year-old boy)

This boy explained that his cisgender friends often failed to understand how painful it was to be misgendered, unlike his nonbinary or binary trans friends, who better empathized with his experience. Another participant shared the emotional exhaustion of repeatedly correcting the same people about their gender. Many of the youths described experiencing "social gender dysphoria," which was triggered by not being recognized for their true gender. This was particularly difficult for those frequently questioned or misgendered, as it heightened their self-awareness of a body that did not align with their gender identity. This constant awareness was described as both emotionally exhausting and energy draining:

It takes so much energy knowing that my voice is too bright, or my face is too feminine, or my binder isn’t tight enough. Like when I talk-, when I raise my hand and say something in class, everybody hears my voice, and that voice is too bright for anyone to believe I’m a boy and those thoughts are in my head 24-7 and really drain all my energy. It often feels like I have no energy left after school and some days I don’t even have the energy to go to school but end up lying in bed all day totally exhausted. (15-year-old boy)

The participants described that increased knowledge among other people led to significant improvements in how they were treated, relieving them from constantly having to explain themselves. With schools taking responsibility for keeping up with knowledge about gender diversity and education, the participants experienced less invalidation and fewer privacy-infringing questions. Teachers knowledgeable about LGBTQ issues were highly appreciated by all participants in the study and education about gender diversity was seen as a crucial factor in shifting peers’ attitudes:

Most people [at school] have known what it is [transgender] but not so much about it. I have had to explain pretty much about it and what it means and yeah, how it works, but-, most of them have been willing to learn, but before I came out in class we had some lessons about LGBTQ-people at school with my natural science teacher—one of the best teachers ever—and I had to defend nonbinary people’s right to exist against half of my class which was rather frustrating, but nowadays they seem better at it. (15-year-old girl)

Some youths felt a strong sense of responsibility also to act as role models and work toward making the world a better place for transgender people, perhaps as a response to the injustices they had faced: *"I want to leave the world a better place than it was when I got here […] I want to be a good role model, that’s my goal."* (15-year-old girl)

#### Reorientation and the power of recognition

The participants who came out as teenagers often began by confiding in a friend, partner, therapist, or online community before telling their parents, unlike those who expressed their gender identities openly from a young age. Coming out relieved the burden of secrecy, providing a sense of connection and reducing feelings of isolation:

It was a relief having told someone, it felt more like-, that is, I had told people on the internet earlier but not anyone who knew me in real life. It felt very good to have someone who knew, and to be able to talk about it with someone […] I didn’t feel so lonely in it anymore. Because before, it was as if I had my thoughts and so on, but I always felt like-, it was me and my thoughts, but it didn’t feel really real until I had told someone in real life. (15-year-old girl)

This participant explained how sharing her identity with someone significant made her experience feel more real and less isolating. While the youths described how some of their parents responded with immediate acceptance, others needed time to process before offering full support. In contrast, siblings often provided the quickest support: *“And my sister really accepted me heaps and she like-, she doesn’t call me my old name because she knows I don’t want to hear it and such things* (13-year-old boy). Siblings may have found it easier to adjust, as they did not carry the same responsibilities as parents. Additionally, some older siblings had more knowledge about gender diversity:*“He was many years older. So, he understood like what it was [transgender] and like: ‘I have a little brother and not a little sister’”* (16-year-old boy).

All the youths described positive emotions and improved well-being after making a full social transition. Being supported both emotionally and practically was a tremendous relief. They found joy in expressing themselves through chosen names, pronouns, and gender expressions:

It feels SO good [to be called the right pronoun]. I feel so very happy and recognized and every time someone does it, I almost always feel pleasantly surprised because it’s like: ‘Wow, this person respects my pronoun’, even if it is someone like my mom. She almost never gets it wrong, and still, when I hear her call me ‘he’ I get so warm inside, kind of happy, and I just feel like-, it’s just so much better (laughs happily). I feel like I’m recognized for who I am. (15-year-old boy)

For those who had not expressed their gender identity before coming out, changing their gender expressions was particularly joyful. This included actions like getting a short haircut, wearing a skirt, or shaving their legs for the first time. Expressing themselves authentically and being recognized by others in alignment with their self-image had profound effects on their mental health and well-being. The participants reported feeling happier, more relaxed, and less anxious or depressed, especially if they had struggled with mental health or gender dysphoria. Many also noted positive behavioral changes, such as improved school attendance and academic performance:” *Since I came out, I started going to school much more often; it just worked much better there”* (15-year-old boy).

The deeply important feeling of gendered belonging often came from being recognized as their true selves. Some participants shared memorable moments of passing as an ordinary girl or boy, as described by this participant:

Once, there were some old ladies in the line in front of me, and one of them happened to stumble into me, and her friend said:’ Watch out, you almost went into him behind you’. That made me happy because that lady saw me as a boy. Yeah, that was fun […] It felt really good. I don’t think I ever got so happy about someone walking into me [laughs]. (15-year-old boy)

Finding support in LGBTQ communities, both online and in real life, was especially meaningful. The participants described these spaces as places where they could let their guard down and enjoy the company of others who shared similar experiences, without constantly thinking or talking about gender identity, or as this girl put it: *“It’s nice to have people who understand you in another way [who are also trans] and who you can be totally open with*” (15-year-old girl). Being “the norm themselves” made them feel relaxed and gave them a sense of belonging. Others found strength by watching or listening to trans role models on platforms like YouTube or podcasts, gaining hope to endure their struggles.

## Discussion

This study explored the lived experiences of nine Swedish transgender youths related to their gender identities within cisnormative contexts. Since many of our findings align with previous research on TGD youth, we use the term TGD when our results are consistent with and can be generalized to a broader TGD population, as supported by prior studies. The findings are summarized below and discussed through the lens of Ahmed’s (2006) Queer Phenomenology and Honneth’s (1995) Theory of Recognition to emphasize existential dimensions, alongside Hochschild’s (1979) concept of “emotional labor”, which sheds light on the participants’ relational efforts.

The inductive thematic analysis identified two main themes. The first main theme, “Disorientation and existential challenges: navigating self-recognition in a non-inclusive world”, highlights the participants’ struggles to balance their emotional and bodily experiences within their cisnormative contexts. They described how limited personal and societal knowledge—or outright denial—of gender diversity complicated their ability to understand and affirm their identities. The second main theme, “Relational responsibility: emotional work to be recognized”, captures the various ways the participants took responsibility for their safety and the emotional efforts they performed to help others recognize their gender. This theme also emphasizes the positive emotional reactions and profound sense of belonging the participants experienced when they were affirmed and recognized for who they are.

The participants’ experiences of navigating the world were shaped by both their own and others’ limited knowledge of gender diversity. Without the language or awareness that being transgender was even a possibility, many of the youths felt out of place. They described feelings of confusion, unease, discomfort, and shame when they struggled to understand or recognize themselves within cisnormativity. Discovering who they were required significant effort at an existential level. Ahmed uses an analogy (Ahmed, 2006) of how horizontal lines on tracing paper disappear when they are in line with the lines on the paper beneath it. Similarly, dominant societal norms often become invisible, unnoticed by those who align with them. In contrast, a vertical line stands out as oblique or "queer," visibly "out of line" compared to the "straight" lines (using terminology that reflects sexual minority perspectives but also applies to gender diversity in a cisnormative context). When participants described feeling *disoriented* (Ahmed, 2006) because they could not see themselves reflected in their gendered contexts, their reactions illuminate their existential experiences of the otherwise invisible, but profoundly felt pressure of not being “in line” with cisnormativity.

The participants described how they constantly had to balance their own needs in relation to their social contexts in trying to create space where they could exist authentically. They described how they insisted, corrected, explained, and educated others, as well as how they chose their coming out carefully to try to facilitate for others to easier embrace their gender identities. At the same time, they sought to protect their close relationships, taking care not to “burden” loved ones with their struggles illustrating the emotional toll of continually negotiating acceptance and recognition. This is in line with previous research (Budge et al., 2018; Catalpa & McGuire, 2018; Kelley et al., 2022; Saltis et al., 2022). These efforts to reorient the gaze of others to see them as they experienced themselves—while at the same time trying not to lose their close ones’ love and support—required a lot of *emotional labor* (Hochschild, 1979). When the participants navigated relations that did not readily recognize, or even denied their gender identities, it was described as annoying and stressful while the opposite rendered feelings of relief, joy and the peaceful feelings of belonging.

Honneth (1995) posits that a supportive environment is essential, as our identity depends on how others see and recognize us. His three modes of recognition—*love*, in the emotionally intimate sphere of family, romantic partners and friends; *solidarity* in the communal sphere of groups, such as at school or in LGBTQ-communities, and *rights* in the legal sphere of society—together form a theory of how social norms shape individual identities and of what is essential to feel valued as human beings. According to Honneth (1995), recognition through love offers the fundamental self-assurance required for later active participation in public life and serves as the structural foundation for all ethical behavior. Interpreted through the lens of Honneth’s theory the misrecognition in the first sphere of love threatened the participants’ sense of self and emotional security and was sometimes even experienced as stressful to the degree of “*no point in living*”, as one of the youths put it. In line with previous research, recognition of TGD youths’ gender identities is vital for their mental health, visible at an existential level as suicidal thoughts (Austin et al., 2022; Russell et al., 2018) versus joyful feelings (Beischel et al., 2022; Skelton et al., 2024), or as existential isolation (Horner et al., 2023) versus feelings of belonging (McGowan et al., 2022). In some cases, the participants described not only a lack of others’ knowledge, so called *epistemic* injustice (Fricker, 2009; Lundberg et al., 2024), but also denial of their gender identities, which implies an *ontic* injustice in not being recognized as existent at all regarding their gender identity. When having their experience denied in this fundamental way it becomes understandable that the participants who had a lot of these kinds of experiences started to question the meaning of living itself.

Even though the participants experienced a lot of support in their families as well as at school they also described experiences that reflected a lack of *solidarity* (Honneth, 1995), threatening their security. Experiences of gender-based bullying, particularly in schools, often resulted in minority stress, as documented in previous studies (Chodzen et al., 2019; Eisenberg et al., 2019). Painful incidents, but also a nagging worry of future exposure, gave way to anxiety, depressive feelings, stomach aches, fatigue, and sleeping disturbances. It also made school attendance and participating in social and physical activities harder. This had possible negative effects on school performance as well as physical health by not getting their needed exercise. Withdrawal could also mean missing out on the essential social support and feeling of belonging shown in research to be the most important resilience-enhancing factors to counteract health effects from minority stress in TGD youth (Austin et al., 2022; Durwood et al., 2021; Tankersley et al., 2021).

Moreover, the concept of microaggressions (Nadal et al., 2012), such as being misgendered daily or asked intimate questions, illustrates the continuous, low-level stress the participants endured. These microaggressions, especially when coming from familiar and essential figures like teachers or peers, were not only hurtful but also exhausting. Other studies have shown that microaggressions can lead to hypervigilance (Bränström, 2019) with negative expectations of not being read correctly, and sometimes even to suicidal thoughts or suicide attempts (Austin et al., 2022) when being largely misrecognized. Moreover, microaggressions seem more frequent than violent acts, as was the case in our study as well as in others (Lundberg et al., 2024; Nadal et al., 2016). Even though more subtle than physical violence, microaggressions have proven to have a profound negative impact on TGD youths’ health (Austin et al., 2022).

The participants described taking responsibility for their own safety by hiding or downplaying their gender identities, to not draw attention to themselves. They also avoided certain contexts to prevent possibly threatening situations, which limited their living space. The concept of emotional labor (Hochschild, 1979) captures not only strategies used in specific stressful situations but also the ongoing coping described by the participants, highlighting a more procedural understanding of coping, in line with previous research (Linander et al., 2019; Lundberg et al., 2024; Tyni et al., 2024a).

The *placement* of responsibility seems like yet another important psychological factor relevant for the consequences on TGD youths’ well-being, as pointed out by Lundberg et al. (2024). For instance, examples in our study showed how blaming oneself for not asserting one’s gender identity enough resulted in feelings of shame and guilt. This illustrates how deeply internalized misrecognition can become, and how social shame is experienced on an individual level. It also made it hard to actively influence situations, as participants experienced themselves being hindered by the shame and guilt. Honneth (1995) suggests that social shame can be mitigated through active protest and collective resistance and mentions the civil right movement as an example of this. Understanding the effects of social shame “pouring down” from legal frameworks and public attitudes to an individual level (Bränström, 2019) is crucial for empowering TGD youths, and vital in combating discrimination and achieving equality. According to Honneth (1995), recognition in the sphere of solidarity is connected to social esteem, where individuals get appreciated for their contributions to society and are being valued by a community. In modern societies, there is a continual struggle for social esteem, he says, dependent on the dominant interpretations of societal goals, where different groups try to elevate the values associated with their way of life. Solidarity is easier to achieve within groups sharing common goals. Therefore, the real challenge, according to Honneth, lies in extending this solidarity to a broader societal context, as it relies on mutual recognition of each other’s unique qualities.

The participants’ struggles to obtain a diagnosis or legal recognition of their gender identities reflected a failure of recognition in Honneth’s (1995) third sphere of *rights*. Being required to provide “*evidence of being trans*”, as one youth put it, to gain the legal recognition and rights that are automatically afforded to cisgender persons was described as a frustrating barrier. For others, the dominating trans narratives, emanating from this medical perspective, was a barrier to recognizing their transgender identity since they did not suffer from intense gender dysphoria or severe mental health issues. The participants’ experiences show that recognition through rights is necessary for TGD youths to be able to, as well as feel worthy of, participating as full members of society. The absence of recognition contributed to the injustices the participants faced. It limited their abilities to understand themselves and limited their legal rights, since gender self-determination it still not a possibility for TGD people in Sweden.

Ahmed argues that it is not only the ones that are “out of line” that can feel disoriented when not recognizing themselves in the world. The mere presence of marginalized individuals can disrupt and disorient those who align with dominant norms, tilting their familiar world view, revealing the constructed nature of these norms (Ahmed, 2006). This was evident in the varied reactions the participants encountered, from mild skepticism to outright hostility. As Ahmed points out, the dominant norms are not simply given, but an effect of an alignment process that either limits or gives access to existential (as well as physically secure) living space depending on how well you align with the majority. There is a pressure that pushes us along certain paths, for example pushing us to align with the others in a cisnormative family or with the dominant norms of society, but at certain “breaking points” we can refuse to inherit or follow the lines given (Ahmed, 2006), just as the participants did when coming out. Despite these challenges, the participants’ gender identity journeys reflected a refusal to conform to cisnormative expectations by asserting themselves through gender expressions, seeking validating contexts also outside their supportive families, seeking gender affirming-care and to get the same legal rights as others. All these active efforts could be seen as attempts to reorient themselves in relation to the cisnormative terrain—a form of active resistance to the cisnormative lines surrounding them.

Gaining knowledge about gender diversity played a pivotal role in helping participants come out and find new ways of being in the world aligning their external lives with their internal sense of self. To support TGD youth, it is essential to address the systemic and relational barriers they face. This includes educating professionals, families, and society about the impacts of minority stress and the importance of recognition in all its forms. By fostering greater awareness and solidarity, we can create a more inclusive world where TGD youth can thrive. Ahmed suggests us all to reflect on *how* we approach moments of disorientation—our own or others—rather than try to define a new (possibly limiting) “*queer orientation*” (Ahmed, 2006, p. 179). Do we as individuals, as communities and societies try to put what seems odd or feels strange “in line” or can we open up to queer moments of deviation and seek support for ourselves, or give support to those diverting from the norm as equal human beings?

### Strengths and limitations

This study has several strengths and weaknesses. One of the weaknesses was the lack of diversity in the sample. All participants came from supportive, white, middle-class families. Additionally, all participants had binary gender identities, despite some variation in their gender assigned at birth, the duration of their gender identification, and the age at which they came out. Including non-binary identities and participants with intersecting experiences, such as low-income backgrounds or experiences of racism, could have provided a more varied and comprehensive under­standing of their experiences.

The main strength of the study is its novelty. It is the first to explicitly discuss and illuminate the existential dimensions of transgender youths internal and intersubjective experiences within a cisnormative society. The ideological context is crucial for understanding TGD youths’ lived realities and have been identified as priorities for further research on LGBTQ people’s experiences (Linander et al., 2019; Lundberg et al., 2024). Therefore, our study contributes valuable data to the field. Furthermore, the integration of interdisciplinary theories with psychological theories enhances previous understandings of TGD youths’ lived experiences from minority stress, micro-aggressions, and coping (Tyni et al., 2024a), highlighting the often-overlooked existential dimensions and continuous every day work involved in their struggles.

The trustworthiness of the findings was enhanced through regular discussions of the analysis with a co-researcher and collaboration within the research group on the final drafts.

#### Learning points

1. **Existential impact of knowledge on gender diversity**: The participants’ experiences highlight the crucial role of knowledge about gender diversity—or the lack thereof—in shaping TGD youths’ ability to orient themselves in the world and find authenticity. The absence of information can lead to profound existential disorientation, adding a new layer of understanding to the challenges faced by TGD youth.
2. **Existential relevance of (mis-)recognition**: The discussion underscores the existential distress and loneliness that arose when the participants were not recognized in their cisnormative contexts. Conversely, when recognized, feelings of joy and belonging are enhanced. This adds a deeper understanding of (mis-)recognition at an existential level.
3. **Emphasis on emotional work**: The study highlights the often invisible, extensive, and continuous emotional labor that TGD youth must engage in to secure vital emotional, communal, and legal recognition for their mental health and well-being. The concept of emotional labor suggests a continuous understanding of coping, contextualizing TGD youths’ experiences within a cisnormative world that often limits their lives.
4. **Reorientation as a form of resistance**: the participants worked to find new paths for authentic living. Their efforts to assert their gender identities through gender expressions, seeking gender-affirmative care, and finding supportive contexts can be seen as forms of active resistance to giving up their internal sense of self in a cisnormative world.

### Implications for future research and practice

This study emphasizes the importance for both professionals and laypeople to understand the existential dimensions of TGD youths’ lived experiences within cisnormativity, the power of (mis-)recognition for their health and wellbeing and the emotional labor they must undertake to create livable space for an authentic life. A holistic perspective, including existential dimensions, is needed to study and support TGD youth, as well as situating their experiences within the broader context of cisnormative societal norms. Such an approach can help shift dominant narratives away from a narrow medicalized discourse, instead emphasizing the resilience and strength TGD youth demonstrate in navigating cisnormativity.

## Supplementary Material

Appendix A Interview guide.docx
