# Supplementary material for: Existential dimensions of the lived experiences of transgender youths 13-16 years in a cisnormative Swedish context
Source: Int J Transgend Health. 2025 Jan 6;27(2):751–66. doi: 10.1080/26895269.2024.2440864 (PMC13015033; doi:10.1080/26895269.2024.2440864)
Supplement: Appendix A Interview guide.docx [file WIJT_A_2440864_SM9365.docx]

**Appendix A. Interview guide**

1. Name

- What is your name?
- Have you ever changed your name? If so, can you tell me about it? (Why? Thoughts/feelings related to name and name change? How did the name come about/who chose it? etc.)

Pronoun

- What do you want me to say about you - he, she, they, or something else?
- Has someone sometimes said the wrong name or thing [pronoun] about you? (If yes, where/who? how did that feel? What did you do?)

1. Gender identity

- What word do you use about your gender? (boy, girl, something else?)
- How does one know that someone is X [the gender the child identifies as] (compared to other gender identities)?
- How do you express your gender – does it show in some way that you are X (describe!)? (How does it feel?)
- Best/worst thing about being X? (how does it feel? How do you cope with X?)
- Do you remember when you first became aware of being X [self-defined gender identity] – can you tell me about it? (how did you notice? Feelings/thoughts?)
- Did you know there are others feeling the same way [gender identity]
- Have you heard the word trans [if not mentioned before]? (where? Who told you? What does it mean?)
- What makes you feel good? (related to gender identity)
- What makes you feel bad?
- How do you cope with it?

1. Social areas

- Did you tell anyone when you knew that you are X – if yes, to whom did you first tell? How did X react? How was that for you (thoughts, feelings)?
- Do they know about your gender identity in preschool/school/leisure activities? If yes, how did it happen (who told? Positive/negative reactions received? How did/do you feel about that?
- What do other people think about or react to you being X? (parents, siblings, relatives, friends, at preschool/school, etc. – describe situations/examples! How does that make you feel?)
- Good/bad incidents? Coping with them?
- How do you cope with X [different negative/positive feelings]? (what do you do if you feel X (examples/situations!)? what makes it feel better?)
- Have you met other children/people who identify as some other gender than their sex assigned at birth? (who, for example, feels they are a boy/girl even though they were called girl/boy at birth? If yes, when, where? Feelings/thoughts/activities?)
